# Host Defense Peptides of Thrombin Modulate Inflammation and Coagulation in Endotoxin-Mediated Shock and *Pseudomonas aeruginosa* Sepsis

**DOI:** 10.1371/journal.pone.0051313

**Published:** 2012-12-13

**Authors:** Martina Kalle, Praveen Papareddy, Gopinath Kasetty, Matthias Mörgelin, Mariena J. A. van der Plas, Victoria Rydengård, Martin Malmsten, Barbara Albiger, Artur Schmidtchen

**Affiliations:** 1 Division of Dermatology and Venereology, Department of Clinical Sciences, Biomedical Center, Lund University, Lund, Sweden; 2 Division of Infection Medicine, Department of Clinical Sciences, Biomedical Center, Lund University, Lund, Sweden; 3 Department of Pharmacy, Uppsala University, Uppsala, Sweden; 4 Medeon Science Park, Malmö, Sweden; 5 European Centre for Disease Prevention and Control (ECDC), Stockholm, Sweden; University of Bern, Switzerland

## Abstract

Gram-negative sepsis is accompanied by a disproportionate innate immune response and excessive coagulation mainly induced by endotoxins released from bacteria. Due to rising antibiotic resistance and current lack of other effective treatments there is an urgent need for new therapies. We here present a new treatment concept for sepsis and endotoxin-mediated shock, based on host defense peptides from the C-terminal part of human thrombin, found to have a broad and inhibitory effect on multiple sepsis pathologies. Thus, the peptides abrogate pro-inflammatory cytokine responses to endotoxin *in vitro* and *in vivo.* Furthermore, they interfere with coagulation by modulating contact activation and tissue factor-mediated clotting *in vitro*, leading to normalization of coagulation responses *in vivo*, a previously unknown function of host defense peptides. In a mouse model of *Pseudomonas aeruginosa* sepsis, the peptide GKY25, while mediating a modest antimicrobial effect, significantly inhibited the pro-inflammatory response, decreased fibrin deposition and leakage in the lungs, as well as reduced mortality. Taken together, the capacity of such thrombin-derived peptides to simultaneously modulate bacterial levels, pro-inflammatory responses, and coagulation, renders them attractive therapeutic candidates for the treatment of invasive infections and sepsis.

## Introduction

Sepsis and septic shock due to systemic bacterial infections are a major cause of mortality in intensive care units and generate high health care costs. Although there is an improvement in standard care procedures, including the use of antibiotics, oxygen, fluid resuscitation and corticosteroids [1], the mortality rate still ranges from 30–50% in patients with septic shock [2], [3]. The opportunistic Gram-negative bacterium *Pseudomonas aeruginosa* is a major pathogen, which can cause both localized and systemic infections [4], [5], e.g. burn wound infections, pneumonia, infections in patients with cystic fibrosis, intra-abdominal infections, chronic ulcers, and sepsis [6]. Because of the emergence of multidrug-resistant *Pseudomonas* strains these infections are a severe problem in hospitals [6]. During septic shock the recognition of bacterial endotoxins (LPS) through pattern recognition receptors [7] induces an initial systemic pro-inflammatory phase characterized by a massive release of cytokines, acute phase proteins and reactive oxygen species. Additionally, activation of proteolytic cascades, like the coagulation and complement system, takes place in combination with impaired fibrinolysis, and consumption of coagulation factors and other mediators [8]–[10]. Many pathways are systemically activated during septic shock, suggesting a significant cross-talk between cellular pro-inflammatory responses, coagulation and the complement systems [11], [12]. The endotoxin-induced upregulation of tissue factor (TF) within the vasculature underlines the dramatic and often detrimental hemostatic disturbances in sepsis [13]–[15] which can lead to organ dysfunctions, organ failure and finally to death.

Antimicrobial peptides are important components of innate immunity [16]–[19]. They significantly contribute to defense mechanisms against invading pathogens at both epithelial surfaces and in blood by being antimicrobial, and also by mediating various biological responses, including chemotaxis, angiogenesis, and anti-endotoxic effects. This multifunctionality has motivated the use of the term “host defense peptides” (HDP) for this group of molecules [20]–[22]. The central role of HDPs in host defense thus makes them interesting candidates in the search for novel treatment options for infections [23]–[25]. Recently, we identified novel endogenous HDPs released from the C-terminus of thrombin by proteolytic cleavage [26]. These fragments are generated during wounding [26] and are antimicrobial against Gram-negative and Gram-positive bacteria, as well as fungi. The prototypic C-terminal peptide of thrombin, GKY25 (GKYGFYTHVFRLKKWIQKVIDQFGE), representative of this class of HDPs, was found to exert anti-inflammatory effects during LPS-shock, and reduced bacterial levels during *Pseudomonas aeruginosa* sepsis. In spite of these promising findings, several important questions dealing with i) the role of the observed direct LPS-peptide interaction *in vitro* for the peptides anti-inflammatory effect *in vivo*, ii) the importance of the antimicrobial effect for the outcome *in vivo*, and iii) presence of possible other peptide mediated actions explaining the beneficial effects *in vivo*, still remained unanswered.

In this study, we therefore set out to determine the immunomodulatory effects of GKY25, but also of the peptide HVF18 (HVFRLKKWIQKVIDQFGE), a shorter fragment that is generated by neutrophil elastase-mediated proteolysis of thrombin [26]. Both peptides abrogated inflammatory responses in LPS-models *in vitro* and *in vivo*, and importantly, GKY25 was found to inhibit the over-activated coagulation response observed during septic shock, leading to normalized coagulation parameters during LPS shock *in vivo*. In animal models of invasive infection with *P. aeruginosa*, GKY25, while demonstrating a modest reduction of bacterial levels, mediated a significant inhibition of the pro-inflammatory response and reduced leakage and fibrin deposition in the lungs. Considering the limited effect on bacterial levels by the peptide, the data highlight the importance of the anti-inflammatory effect during bacterial infection *in vivo*. Furthermore, the data disclose a previously unknown role of thrombin-derived peptides in the control of coagulation, based on blocking of both TF-expression and contact activation. Finally, the results imply new treatment possibilities for sepsis, based on combined inhibition of bacterial growth and pro-inflammatory cytokine responses, joined with a simultaneous blocking of excessive activation of coagulation pathways initiated during sepsis.

## Materials and Methods

### Ethics statement

The use of human blood was approved by the Ethics Committee at Lund University, Lund, Sweden (Permit Number: 657-2008). Written informed consent was obtained from the donors. The animal experiments were conducted according to national guidelines (Swedish Animal Welfare Act SFS 1988:534) and were approved by the Laboratory Animal Ethics Committee of Malmö/Lund, Sweden (Permit Numbers: M75-10, M227-10, M228-10).

### Peptides

The thrombin-derived peptides GKY25 (GKYGFYTHVFRLKKWIQKVIDQFGE) and HVF18 (HVFRLKKWIQKVIDQFGE), as well as the control peptide WFF25 (WFFFYYLIIGGGVVTHQQRKKKKDE), were synthesized by Biopeptide Co., (San Diego, USA). The purity (>95%) of these peptides was confirmed by mass spectral analysis (MALDI-ToF Voyager).

### Bacteria and cells

The clinical isolate *Pseudomonas aeruginosa* 15159, *Escherichia coli* ATCC 25922 and *Pseudomonas aeruginosa* ATCC 27853 were purchased from the American Type Culture Collection (ATCC, Rockville, MD) or obtained from the Department of Bacteriology, Lund University Hospital, Sweden. The mouse macrophage cell line RAW 264.7 was obtained from the American Type Culture Collection (ATCC, Rockville, MD). RAW 264.7 cells were cultured in Dulbecco's modified Eagle medium (DMEM; PAA-Laboratories) supplemented with 10% (v/v) heat-inactivated fetal bovine serum (FBS) (Invitrogen) and 1% (v/v) Antibiotic-Antimycotic solution (AAS) (Invitrogen). Human peripheral blood mononuclear cells (PBMNCs) from healthy donors were isolated from fresh heparinized or citrated blood by Lymphoprep™ (ρ = 1.077 g/mL; Axis-Shield, Norway) density centrifugation at 700 g for 20 min. PBMNCs were collected in RPMI 1640 (PAA-Laboratories). For FACS analysis, monocytes were purified using anti-CD14 coated microbeads (Miltenyi Biotec GmbH, Germany). Monocyte purity was >96%.

### Animals

For all animal experiments C57BL/6 mice, purchased from Charles River or the Animal facility Lund University were used. The animals were housed under standard conditions of light and temperature and had free access to standard laboratory chow and water.

### LPS models

#### Nitrite assay

RAW 264.7 cells (3.5×10^6^/ml) in phenol red-free DMEM supplemented with 10% (v/v) FBS and 1% (v/v) AAS were seeded in 96-wells tissue culture plates (Nunc). Following 20 h of incubation to allow adherence, cells were washed and stimulated with either 10 ng/ml *E. coli* (0111:B4) or *P. aeruginosa* LPS (serotype 10), (Sigma-Aldrich, approximate 500.000 endotoxin units/mg) together with or without various concentrations of GKY25, HVF18, and WFF25. The level of nitrite oxide (NO) in culture supernatants was determined after 20 h incubation using the Griess reaction as described previously and is presented as Nitrite (µM) [27].

### TNF-α assay

Whole human lepirudin-treated blood was incubated with 10 ng/ml *E. coli* (0111:B4) LPS, with or without the peptides GKY25 or HVF18 for 6 h on rotation at 37°C. Plasma was obtained and stored at −20°C. The TNF-α level was determined using a human TNF-ELISA Kit (Invitrogen).

### Coagulation assays

All clotting times were analyzed using a coagulometer (Amelung, Lemgo, Germany). For determination of prothrombin time (PT) and thrombin clotting time (TCT), a thromboplastin reagent (Trinity Biotech) and Thrombin reagent (Technoclone) were used, respectively. Hundred µl of fresh citrate plasma, together with indicated concentrations of GKY25, HVF18 or WFF25 were pre-warmed for 60 sec at 37°C before clot formation was initiated by adding 100 µl of clotting reagent. To record the activated partial thromboplastin time (aPTT), 100 µl of a kaolin-containing solution (Technoclone) was added to the plasma-peptide mix and incubated for 200 sec before clot formation was initiated by adding 100 µl of 30 mM fresh CaCl_2_ solution. Alternatively, 1×10^6^ hPBMNCs/ml in RPMI 1640 were stimulated with 100 ng/ml *E. coli* (O111:B4) LPS with and without GKY25 or HVF18 overnight on rotation at 37°C. Cells were washed and resuspended in 100 µl PBS. One hundred microliter of fresh human citrate plasma were reconstituted with 100 µl of fresh 30 mM CaCl_2_ solution and pre-warmed for 60 sec. The clot formation was started by the addition of hPBMNC. The same procedure was used to determine clotting times for whole blood cells from 500 µl of blood.

Thrombin/Antithrombin complexes were determined by using a mouse TAT ELISA-Kit (Uscn Life Science Inc.).

### Flow cytometry

Human monocytes (1×10^6^ c/ml) in RPMI 1640 containing 10% (v/v) FBS and 1% (v/v) AAS were stimulated with 100 ng/ml *E. coli* (OIII:B4) LPS with or without various concentrations of GKY25 or HVF18 over night. Cells were washed with PBS containing 0.2% BSA and resuspended in the PBS, 0.2% BSA solution followed by incubation with a monoclonal FITC-anti-human TF antibody (clone VD8, American Diagnostics) or FITC-IgG1 (BD Biosciences), PE-anti-CD14 or PE-IgG1 (BD Biosciences) for 30 min on ice. Samples were analysed using a FACS Calibur flow cytometer (BD Biosciences) and the Cell Quest and FlowJo software (BD Bioscience).

### LPS animal model

Male C57BL/6 mice (8 weeks, 21+/−5 g) were injected intraperitoneally (i.p.) with 18 mg *E. coli* 0111:B4 LPS or 36 mg *P. aeruginosa* LPS (Sigma) per kg of body weight. Thirty minutes after LPS injection 0.5 mg GKY25, HVF18, or WFF25 (10 mM Tris, pH 7.4) or buffer alone (control) were injected i.p. into the mice. Status and weight were daily monitored for seven days. Mice showing the defined and approved endpoint criteria (immobilization and shaking) were sacrificed by an overdose of isoflurane (Abott) and counted as non-survivors. For scanning electron microscopy (SEM) and histology analyses, mice were sacrificed 20 h after LPS challenge, and lungs were removed and fixed. For determination of cytokine levels in mouse plasma, animals were sacrificed 8 h and 20 h after LPS injection. The blood was collected immediately by cardiac puncture.

### Animal infection model

*P. aeruginosa* 15159 bacteria were grown to mid-exponential phase (OD_620_∼0.5), harvested, washed twice in PBS, and diluted in PBS to 2–5×10^9^ cfu/ml. Hundred microliter of this bacterial suspension were injected i.p. into male C57BL/6 mice (8–9 weeks, 21+/−5 g). After various time points, 0.5 mg GKY25 or buffer alone (control) was injected subcutaneously (sc) into the mice. In order to study bacterial dissemination to target organs, spleen, liver and kidney were harvested in PBS, placed on ice, homogenized, and subsequently colony-forming units were determined. To assess survival, the mouse status was monitored regularly and mice reaching the defined endpoint were sacrificed by an overdose of isoflurane and counted as non-survivors.

### Determination of platelet numbers

The number of platelets in mouse blood (anti-coagulated with EDTA) taken by cardiac puncture was determined and analyzed using the VetScan HM5 System (TRIOLAB).

### Cytokine assay

The level of IL-6, IL-10, MCP-1, IFN-γ, and TNF-α were assessed either in cell culture supernatants from RAW264.7 cells or murine plasma using the Mouse Inflammation Kit, (Becton Dickinson AB) according to the manufacturer's instructions.

### Histochemistry

Organs were collected 20 h after LPS injection and were immediately fixed in 4% formaldehyde prior to paraffin embedding and sectioning. Sections were stained with Mayers Haematoxylin (Histolab AB) and Eosin (Merck).

### Scanning electron microscopy

For scanning electron microscopy, lungs were collected at 12 or 20 h after injection of bacteria or LPS, respectively. Samples were fixed in 2.5% (v/v) glutaraldehyde in 0.15 M sodium cacodylate buffer, pH 7.4, over night at room temperature. Specimens were washed with cacodylate buffer, and dehydrated with increasing amounts of ethanol from 50% (v/v) to absolute ethanol. Next, the specimens were subjected to critical-point drying in carbon dioxide, with absolute ethanol as intermediate solvent, mounted on aluminum holders, sputtered with 30 nm palladium/gold and examined in a JEOL JSM-350 scanning electron microscope. To quantify pulmonary lesions, lung samples from 30 different fields covering an entire lung section were made, and the percentage of fibrin deposits and fields exhibiting hemorrhage were determined. [28]

### LPS binding

One, 2, and 5 µg of peptides were bound to nitrocellulose membranes (Hybond-C, GE Healthcare Bioscience). After blocking for 1 h with BSA, the membranes were incubated for 1 h with ^125^I-labeled *E. coli* LPS (40 µg/ml). Subsequently, membranes were washed three times, before binding was visualized using a Bas 2000 radioimaging system (Fuji). Unlabeled heparin (6 mg/ml) was used for competition of binding.

### Statistics

Values are shown as mean with SEM. In the *in vitro* assays n indicates the total number of independent experiments performed. In the *in vivo* experiments n stands for the total number of animals used in two to three independent experiments. For statistical evaluation of two experimental groups the Mann-Whitney U-test was used and for comparison of survival curves the log-rank test with *p-<0.05, **<0.01 and ***p<0.001.

## Results

### GKY25 and HVF18 dampen the inflammatory response *in vitro*

**Figure 1:**
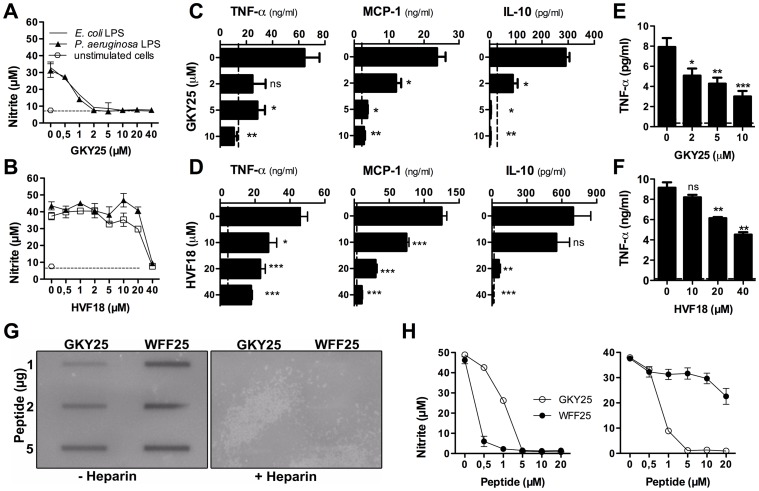
Thrombin-derived peptides modulate the cytokine response to LPS *in vitro.* (A and B) Measurement of nitrite release of RAW 264.7 macrophages stimulated with 10 ng/ml *E. coli* or *P. aeruginosa* LPS in combination with the indicated concentrations of GKY25 and HVF18 (n = 3). (C and D) RAW 264.7 cells were stimulated with 10 ng/ml *E. coli* LPS and cytokines were analysed in the cell supernatants (n = 3). (E and F) Human blood was treated with 10 ng/ml *E. coli* LPS alone, or with LPS and GKY25 (E) or HVF18 (F). After 6 h of incubation TNF-α was determined in plasma by ELISA (n = 3). (G) Binding of GKY25 and WFF25 to ^125I^-labeled *E. coli* LPS using a slot blot assay. (H) Stimulation of RAW 264.7 cells by LPS and the effects of peptides. Left panel: LPS was incubated with GKY25 or WFF25, followed by addition of serum and incubation with the cells. Right panel: Standard procedure as in A. Peptides were added to cells pre-incubated briefly with LPS in DMEM with 10% FBS (n = 3).

In a previous study it was shown that both C-terminal thrombin-derived peptides GKY25 and HVF18 bind to LPS [26]. Initial results using a mouse macrophage model, showed that 2 µM GKY25 completely eliminated the LPS-induced nitrite oxide release from the cells, while 20–40 µM of the shorter fragment HVF18 was required for complete inhibition (Figure 1, A and B). In addition, GKY25 and HVF18 dose-dependently reduced the release of TNF-α, the monocyte chemotactic protein-1 (MCP-1), and IL-10 (Figure 1, C and D). To further explore the effects of GKY25 and HVF18 in a more physiologic environment, the effects of the peptides were evaluated in whole blood. As shown in Figure 1E and 1F, both peptides significantly reduced the TNF-α release in human blood stimulated with *E. coli* LPS. Similar to the above results with mouse macrophages, a higher concentration of HVF18 was required for the anti-inflammatory effect. In summary, these data confirm, as well as extend previous observations into a human system, demonstrating that both the endogenously released HVF18 and the related peptide GKY25 significantly inhibit pro-inflammatory responses *in vitro* and *ex vivo*.

### The anti-inflammatory effects of GKY25 are not solely dependent on LPS binding

To investigate to which extent the above determined effects are i) sequence specific and ii) dependent on direct interactions with LPS, we used the specifically designed peptide WFF25 (WFFFYYLIIGGGVVTHQQRKKKKDE). This peptide has the same amino acid composition as the endogenous helical and amphipathic GKY25, but with the amino acids sorted after hydrophobicity in order to eliminate sequence dependence and create an amphipathic linear structure. WFF25 displays LPS-binding properties similar to those previously described for hydrophobically tagged linear cationic peptides [29]. Thus, direct measurements of LPS binding yielded that WFF25 bound 750 and 750 nmol/mg LPS in Tris and Tris, 0.15 M NaCl, respectively, whereas the corresponding values for GKY25 were 550 and 337.5 nmol/mg LPS (Malmsten M. et al., unpublished results). Slot blot assays using ^125^I-labeled *E. coli* LPS and the peptides WFF25 and GKY25 illustrated the affinity of both peptides to LPS (Figure 1G). The interaction with LPS was completely blocked by heparin. In order to further explore the importance of this direct LPS-binding, the anti-inflammatory effects of the two peptides were analysed in two experimental setups using mouse macrophages. In one experiment, the peptides were incubated together with LPS *before* addition to serum and cells (Figure 1H, left panel), in another setup, the peptide was added to cells *after* addition of LPS to the serum (Figure 1H, right panel). The results showed that in contrast to GKY25, the peptide WFF25, in spite of its LPS-affinity was unable to block the LPS-induced response in cells pretreated with LPS in serum. Thus, the data indicate that although LPS-binding is a prerequisite for the anti-endotoxic effect of GKY25, this is not the sole factor underlying the anti-inflammatory action of the peptide. Moreover, experiments demonstrated that mouse macrophages, pre-treated with GKY25 and thereafter washed, remained unresponsive to LPS and that TAMRA-labeled GKY25, but not TAMRA-WFF25, showed significant binding to mouse and human monocytes (Kalle, manuscript in preparation). Further, GKY25 also reduced the activation of mouse macrophages stimulated with TNF-α, zymosan, or ODN1826 (Figure S1, Materials and Methods S1). Taken together, these results indicate that GKY25 acts via additional mechanisms dependent on direct interactions with macrophages.

### GKY25 and HVF18 modulate coagulation

**Figure 2:**
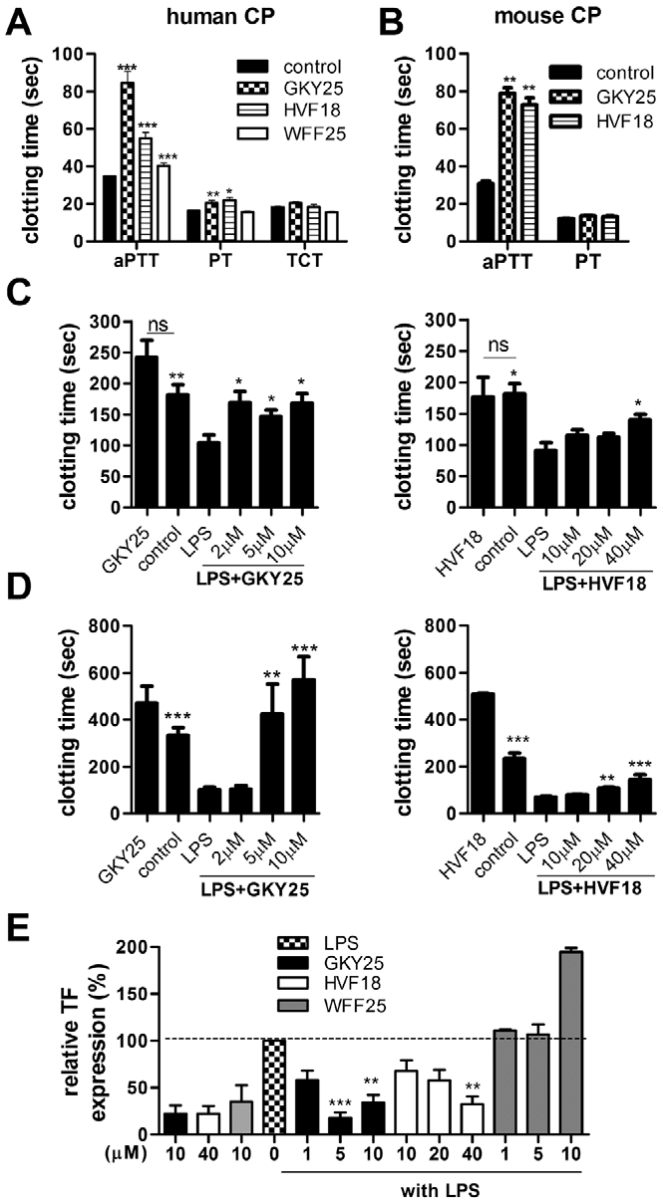
Thrombin-derived peptides influence the coagulation system. (A+B) The activated partial thrombin time (aPTT), prothrombin time (PT) and thrombin clotting time were determined after addition of buffer (control) or 20 µM of GKY25, HVF18 or WFF25 to human citrate plasma (CP) or 50 µM peptide in mouse CP (n = 3). (C) Whole blood was incubated with 10 ng/ml *E. coli* LPS with or without the indicated peptides at different concentrations. The clotting reaction in recalcified human CP was initiated by adding the blood cells. (Significance relative the LPS-treated sample is presented. n = 3) (D) Human monocytes were stimulated with 100 ng/ml *E. coli* LPS with or without the indicated peptides. The clotting reaction was initiated by adding LPS- and/or peptide-treated monocytes to human CP supplemented with CaCl_2_. (Significance relative the LPS-treated sample is presented. n = 3) (E) FACS analysis of TF expression on human CD14-positive monocytes treated with peptides and/or LPS (control; sodium citrate buffer, GKY25 only; indicates 10 µM peptide, HVF18 only; 40 µM peptide; n = 3).

Excessive activation of the clotting cascade via TF-mediated intravascular coagulation and bacteria-induced contact activation contributes to the detrimental effects observed during sepsis and septic shock [30]. Therefore, we investigated possible effects of GKY25 and HVF18, as well as the control peptide WFF25 on coagulation pathways. Analysis of peptide effects on the activated partial thromboplastin time (aPTT) and prothrombin time (PT) showed that GKY25 and HVF18 mainly impaired the intrinsic pathway (aPTT) of coagulation in human and mouse plasma *in vitro* (Figure 2A and B, Figure S2). The peptide WFF25 demonstrated significantly less prolongation of aPTT, particularly at 20 µM, when compared with GKY25 (p<0.0001), and the concentration dependent effects were similar to those observed for the shorter peptide HVF18 (Figure S2). Furthermore, none of the peptides affected thrombin-induced fibrin network formation, as judged by the thrombin clotting time (TCT). (Figure 2A). Next, we investigated whether peptide treatment can interfere with endotoxin-mediated activation of the coagulation cascade in whole blood. As seen in Figure 2C, LPS promotes faster clotting which was significantly reduced by GKY25 and to a lesser extent by HVF18. Notably, GKY25 almost restored the clotting time to normal (control) values at concentrations similar to the physiological concentration of the holoprotein, thrombin (≈1.5 µM). Physiologically, the up-regulation of tissue factor (TF) on the monocyte cell-surface is the main initiator of blood clotting in response to infection and LPS-challenge [31], [32]. We therefore determined the effect of the peptides on TF-mediated coagulation. The results showed that both GKY25 and HVF18 inhibited LPS-induced coagulation in a dose-dependent manner (Figure 2D). Moreover, 5 µM of GKY25 restored the coagulation time to control levels. To confirm that the observed peptide effects were due to reduction in TF expression, FACS analyses using antibodies against TF were employed. Both GKY25 and HVF18 dose-dependently reduced TF expression, in contrast, the control peptide WFF25 did not demonstrate such effects (Figure 2E, Figure S3). Taken together, these results show that GKY25 and HVF18 inhibit contact activation and modulate LPS-induced TF-mediated coagulation, the latter effect of importance for the observed anti-coagulative actions *ex vivo*.

### GKY25 and HVF18 exert immunomodulatory effects *in vivo*

**Figure 3:**
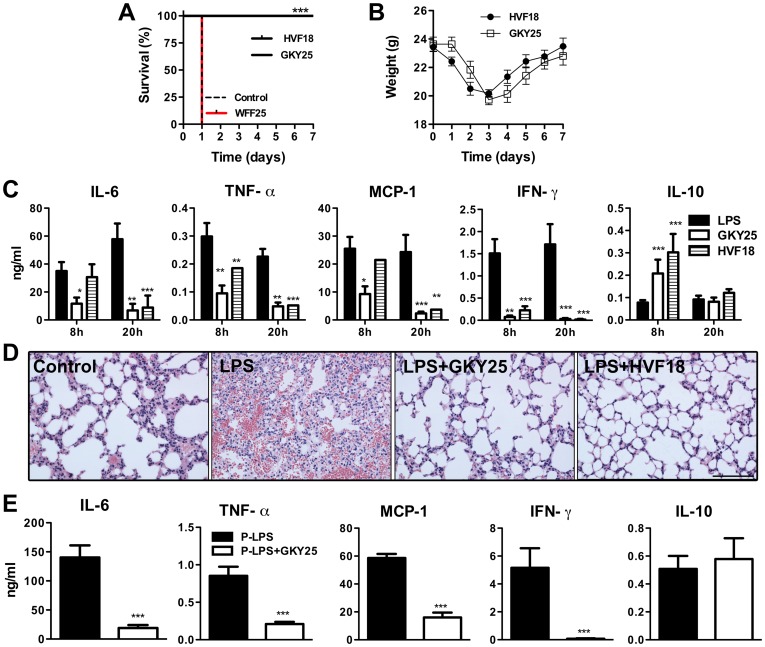
Anti-inflammatory effects of thrombin derived peptides *in vivo*. (A–D) Septic shock in C57BL/6 mice was induced by i.p. injection of 18 mg/kg *E. coli* LPS. Thirty minutes after LPS injection, GKY25, WFF25, HVF18 (0.5 mg/mouse) or buffer were administered i.p. (A) Survival was followed for 7 days (n = 10–19/group (p<0.0001)). (B) Weight of the surviving animals. (C) Cytokines measured in blood from animals sacrificed at 8 h or 20 h after LPS injection (8 h: n = 8–12/group; 20 h: n = 10–14/group). (D) Light microscopy pictures (20× magnification, scale bar: 100 µm) of representative mouse lung tissue from control mice (buffer only), LPS and buffer or LPS and peptide treated mice 20 h after LPS injection. (E) C57BL/6 mice were injected i.p. with 36 mg/k*g P. aeruginosa* LPS followed 30 minutes later by i.p. administration of 0.5 mg of GKY25. After 20 h mice were sacrificed and cytokine levels in blood were determined (n = 8–10/group).

Given the observed dual effects on cytokine responses and coagulation, the *in vivo* efficiency of the thrombin-derived peptides was evaluated in a mouse model of endotoxin-induced shock (Figure 3). A dramatic improvement in survival rate of the animals was seen after treatment with either GKY25 or HVF18, but not with WFF25 (Figure 3A). The GKY25 and HVF18 treated animals fully regained their weight after seven days (Figure 3B). Analyses of cytokines 8 h and 20 h after LPS injection showed significant reductions of pro-inflammatory cytokines IL-6, IFN-γ, TNF-α, and MCP-1, whereas a transient increase in the anti-inflammatory IL-10 was observed for both peptides after 8 h, but not after 20 h (Figure 3C). In contrast, the peptide WFF25 failed to significantly reduce the cytokine levels after 20 h (Figure S4). Histological analyses of the lungs from LPS-treated animals demonstrated a significant inflammatory infiltrate and increased pulmonary leakage of proteins and red blood cells (Figure 3D, Figure S5, Materials and Methods S2), while lungs of GKY25- and HVF18-treated animals showed significantly less of these LPS-induced pathological effects. Comparable cytokine reductions after treatment with GKY25 were seen in a similar shock model using *P. aeruginosa* LPS (Figure 3E). Importantly, treatment with peptides alone (in the absence of LPS) did not induce cytokine release (Table S1). In conclusion, the results from these *in vivo* studies demonstrate that both thrombin-derived peptides display significant anti-inflammatory effects *in vivo*, leading to reduced inflammation and leakage in the lungs, as well as survival of the mice. Considering that GKY25 was more potent in several of the above experiments, this peptide was selected for further *in vivo*-related work.

### Modulation of coagulation *in vivo*

**Figure 4:**
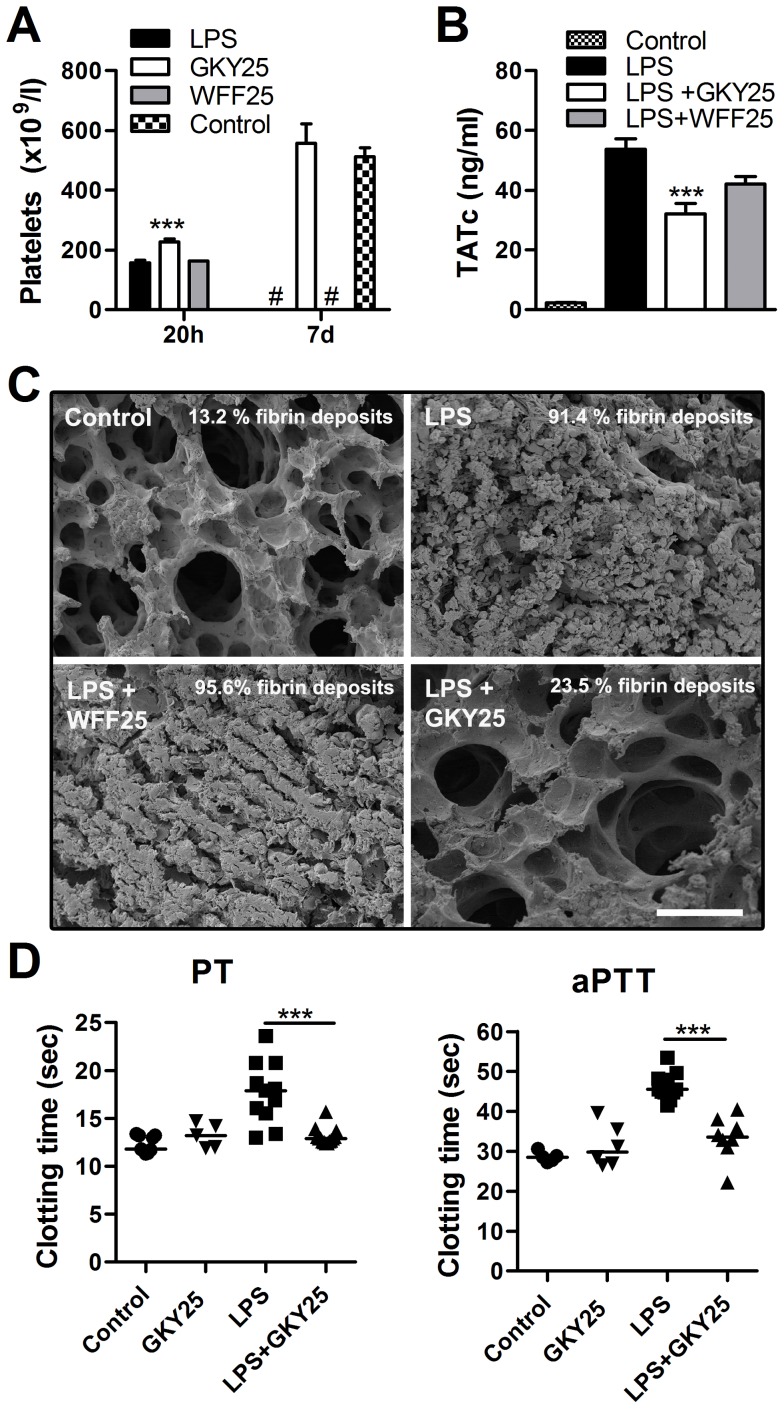
Modulation of coagulation *in vivo*. (A–D) C57BL/6 mice were injected i.p. with 18 mg/kg *E. coli* LPS to induce an endotoxin mediated shock followed by treatment with buffer, GKY25 or WFF25 (0.5 mg/mouse). (A) Animals were sacrificed after 20 h or 7 days and platelet numbers were determined (n = 12–20/group) (#: indicates no animals reached that time point, see Fig. 3A) (B) Quantification of thrombin/antithrombin complexes by ELISA (n = 13–20/group). (C) Comparison of lung lesions in sections 20 h after LPS injection. Scanning electron micrographs show representative mouse lung sections (scale bar: 50 µm). (D) Prothrombin time (PT) (n = 9–14/group) and activated partial thromboplastin time (aPTT) (n = 5–11/group) were measured in citrate plasma isolated from mice 20 h after injection of indicated substances. (***p<0.001).

Previous studies have shown that thrombocytopenia is as an important indicator for the severity of sepsis and disseminated intravascular coagulation [33]. Therefore, we measured platelet numbers in blood of LPS-injected mice treated with or without GKY25 at 20 h, and 7 days after LPS injection. WFF25 was used for comparison. Only GKY25-treated animals showed significantly increased platelet numbers at 20 h, indicating that the peptide reduced platelet consumption in this endotoxin-model (Figure 4A). The levels were completely normalized in the survivors after seven days. Furthermore, GKY25 reduced the levels of prothrombin fragments 1+2 (F1+2), a marker for thrombin formation by 34.8%±6 (n = 5–8/group; P<0.05), and correspondingly, thrombin/anti-thrombin complexes (TATc), were also significantly reduced after treatment with the peptide (Figure 4B). Activated thrombin converts fibrinogen to fibrin, which accumulates within the lungs impairing lung function and gas exchange. Therefore, microvascular fibrin deposits were quantified by scanning electron microscopy of lung sections from mice subjected to LPS-induced shock after treatment with or without the peptides GKY25 or WFF25. Additional analyses included buffer and peptide, respectively. In animals subjected to LPS, significantly increased amounts of fibrin deposits within the lung were observed (Figure 4C). The levels were reduced after treatment with GKY25, and as above, WFF25 showed no such effects (Figure 4C). Moreover, the lung morphology of animals treated with GKY25 resembled untreated mice (Figure S6A).

In response to LPS challenge, the coagulation cascade is activated, leading to an excessive activation of the coagulation system, followed by consumption of coagulation factors in the blood resulting in prolonged clotting times [34]. In line with this, LPS-injected mice showed a reduced clotting capacity and exhibited prolonged aPTT and PT times in their plasma. Treatment with GKY25 however, resulted in a normalized clotting function, as evidenced by coagulation times comparable to those observed for control mice (Figure 4D). Hence, these data showed that the peptide, by blocking LPS-induced coagulation, reduced the excessive consumption of coagulation factors in this animal model. Injection of GKY25 alone did neither affect PT nor aPTT times (Figure 4D). Likewise, administration of a higher peptide dose (1 mg of peptide given twice) yielded no increase in platelet counts, PT, or aPTT times (Figure S6B and C), and no bleeding was observed in the lungs of the animals (not shown). Taken together, the results from these experimental models demonstrate that GKY25 mainly inhibits the excessive, LPS-induced, coagulation response during septic shock *in vivo*.

### GKY25 shows therapeutic potential in a mouse model of *Pseudomonas aeruginosa* sepsis

**Figure 5:**
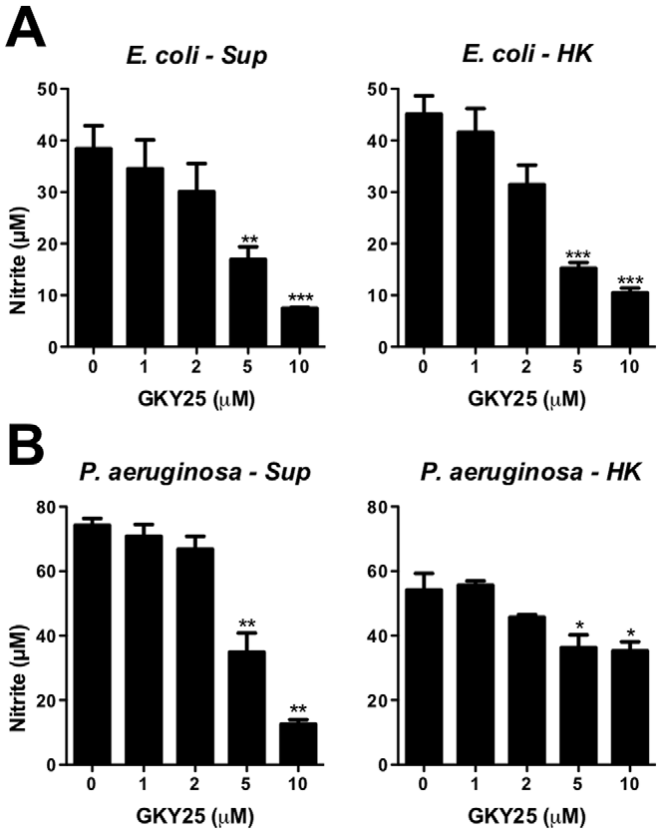
GKY25 modulates NO release in response to bacterial products. RAW 264.7 macrophages were stimulated with *E. coli* (A) or *P. aeruginosa* (B) culture supernatants (Sup) or heat killed (HK) bacteria in the presence of GKY25 and NO release was determined using the Griess reaction.

**Figure 6:**
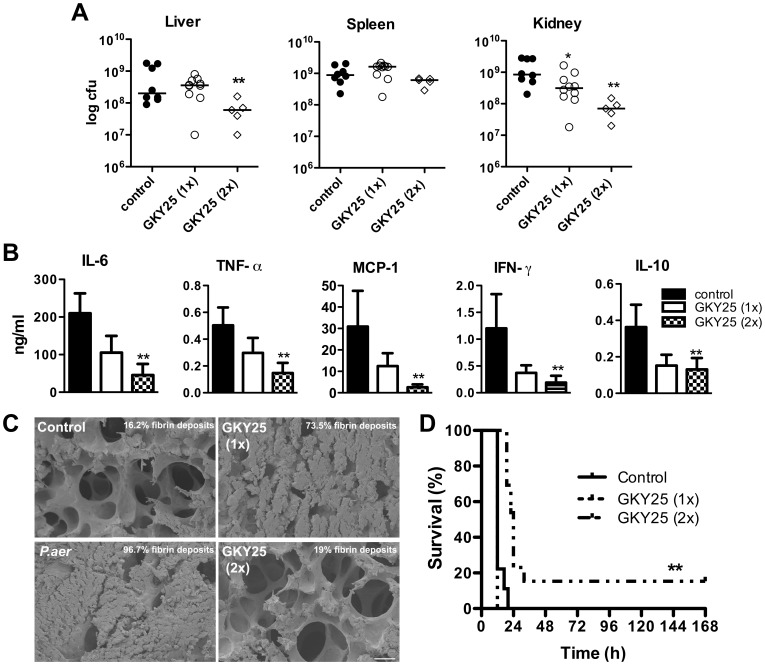
Effects of GKY25 during *P. aeruginosa* sepsis. Mice were infected with *P. aeruginosa* (5×10^9^ cfu/ml) i.p. followed by administration of buffer or GKY25 (0.5 mg) sc. after 1 h (1×), or 1 and 7 h (2×) after infection. (A–C) Mice were sacrificed 12 h after infection. (A) Analysis of bacterial counts in the indicated organs (n = 5–10/group). (B) Cytokine analyses in plasma (n = 8–12/group). (C) Scanning electron micrographs of representative mouse lung tissues. Non-infected mice were used as negative control (scale bar: 20 µm). (D). Survival was monitored for 7 days (n = 9–13/group; **p<0.01).

During infections, bacteria and bacterial products rather than pure LPS, stimulate the host cells. Therefore, we first investigated whether GKY25 can modulate the response to bacterial culture supernatants and heat-killed bacteria in a macrophage model *in vitro*. The results showed that GKY25 significantly reduced NO release of RAW macrophages stimulated with either *E. coli* or *P. aeruginosa* components (Figure 5, A and B), although higher peptide concentrations were required in this model compared to pure LPS. Given the above results, we decided to further explore the potential therapeutic effect of GKY25 in a mouse model of *P. aeruginosa*-induced sepsis. Initial studies with a low infective dose (Figure S7A) showed that the bacterial load increased between 4 to 12 h in all organs analyzed (spleen, kidney and liver). Treatment with GKY25 marginally reduced bacterial loads, and the reductions were only statistically significant after 12 h for liver and spleen. However, a reduction in the levels of pro-inflammatory cytokines IL-6, IFN-γ, TNF-α and MCP-1 was observed, particularly after 12 h (Figure S7B). Platelet counts revealed less platelet reduction at 12 h (Figure S7C), which is comparable to the results obtained on coagulation in the LPS-model. Moreover, similar findings on cytokine modulation after peptide treatment were also demonstrated using a higher infective dose of *P. aeruginosa* (Figure S8). Higher levels of cytokines in *P. aeruginosa* infected animals were observed, most likely reflecting the higher bacterial levels (data not shown). Based on these initial results using a single dose of peptide, the effects of single as compared to repeated administration of GKY25 were evaluated in the *P. aeruginosa* sepsis model. In Figure 6A, we observed that repeated treatment yielded a moderate reduction of bacterial numbers (cfu) in the different organs. This relative modest inhibition of bacteria contrasted to the observed significant reduction of cytokines in the blood of the animals (Figure 6B) and a marked reduction of pulmonary leakage and fibrin deposition in the lungs of the animals (Figure 6C). Notably, treatment with GKY25 significantly reduced pulmonary fibrin deposits, and the effect was particularly noted in mice treated twice with the peptide. Furthermore, repeated administration of GKY25 resulted in a significant delay of septic symptoms and increased the survival rate (Figure 6D). Taken together, although antimicrobial effects were observed, particularly after repeated administration of GKY25, the results indicate that the major effects of GKY25 *in vivo* are dependent on the immunomodulatory and anti-coagulative actions of the peptide.

## Discussion

Considering the multiplicity of inflammatory mechanisms activated during sepsis, and the lack of clinical efficacy in treatment studies targeting specific individual pathways, it has recently been proposed that modulation of the host response rather than blocking one pathway might be beneficial in order to avoid or dampen dangerous complications during the development of sepsis [3], [35]–[40]. In this perspective, our data support the concept that simultaneous modulation of different aspects of the innate response, such as Toll-like receptor (TLR) mediated pathways, and coagulation can lead to an improved outcome during sepsis. Apart from demonstrating these effects *in vitro*, our results obtained from the mice models, in which the peptides were administered after LPS, indicate the ability of GKY25 and HVF18 to protect animals from acute septic shock by reducing vascular leakage, overactivation of coagulation and inflammation as observed by a substantial decrease in a range of pro-inflammatory cytokines, e.g. IL-6 and TNF- α. The study thus presents the novel concept that a human endogenous peptide, such as the thrombin-derived C-terminal peptide described here is multifunctional and not only participates in defense against bacteria, but also modulates coagulation and inflammation, a capacity which may be utilized therapeutically in order to compensate for, and normalize, the excessive inflammatory and coagulative response seen in sepsis.

Considering the role of a direct peptide LPS-interaction *in vivo*, our results with WFF25, which also binds LPS with high affinity *in vitro*, challenge the view that the peptide GKY25 functions solely via extracellular endotoxin scavenging, as described for several other antimicrobial peptides [41]. Therefore, the data suggest that a direct LPS binding, although present, may not explain the complete mode of action of GKY25, but indicate that additional mechanisms must be present mediating the activity of this peptide. As mentioned above, recent data reveal that GKY25 binds to macrophages and monocytes (Kalle et al., in preparation), and experiments are now directed at defining the mode of action on TLR-receptors, possible interactions with cell-membranes as well as extracellular receptors, and related downstream signaling by GKY25 and related thrombin-derived HDPs. Furthermore, previous findings, showing that an internal fragment of GKY25 of 12 amino acids (VFRLKKWIQKVI) binds LPS but exerts no anti-endotoxic effects on macrophages [42], along with the observation that the thrombin-derived C-terminal region has a rather unique anti-inflammatory capacity among several other antimicrobial (and hence, LPS-interacting) C-terminal sequences of S1-peptidases [43], is also compatible with an additional mode of action, independent of the LPS-interaction. In this context, it should be mentioned that analogously to the observations in this work, many HDPs which have been found to display extracellular LPS-binding and/or LPS-disaggregating properties [41] also have been shown to exhibit effects on cell-signaling pathways. For example LL-37 exerts multiple immunomodulatory activities including modulation of the TLR-mediated inflammatory response [44], and recent findings show that human β-defensin affects the activity of LPS-stimulated macrophages by inhibiting pro-inflammatory pathways associated with MyD88 and TRIF [45]. The exact structural prerequisites of GKY25 enabling the immunomodulatory and related anti-coagulative effects clearly remain to be investigated. However, it is notable that the shorter HVF18 is less potent than GKY25, suggesting the presence of interactions, which are length-dependent and therefore influenced by helix formation of these peptides [42]. This reasoning is compatible with the strongly reduced effects observed for WFF25 compared to GKY25, since the former peptide, in contrast to the endogenous sequence GKY25 [26] is unable to form a helix on LPS interaction (Malmsten et al., in preparation).

From a therapeutic viewpoint, it is notable that apart from the significant beneficial effects in LPS mouse models, GKY25 also counteracts *P. aeruginosa* sepsis, with a concomitant and pronounced reduction in pro-inflammatory cytokine levels and vascular leakage. In comparison to a single dose of GKY25, repeated peptide administration yielded a relatively modest reduction in bacterial numbers, which contrasted to the marked inhibition of pro-inflammatory responses, an observation clearly differentiating between directly antimicrobial and modulatory effects on the immune and coagulation systems for this peptide, and illustrating the relative importance of the immunomodulatory and anti-coagulative effects *in vivo*. The fact that the peptide was given subcutaneously, whereas the bacteria were injected intraperitoneally (avoiding compartmentalization of bacteria and peptide together) further suggests a therapeutic potential of systemic peptide treatment. The observation that repeated peptide administration significantly improved the survival rate was not unexpected. However, both proteolytic degradation of GKY25 by bacterial proteases, analogous to those reported for the peptide LL-37 [46], [47], and scavenging through binding of the positively charged GKY25 to negatively charged serum proteins and tissue components cannot be excluded, thereby leading to consumption of free and intact GKY25, explaining a transient pharmacokinetic potency.

For both LPS-induced shock and *P. aeruginosa*–induced sepsis, regulation of excessive cytokine levels is regarded as a relevant target in sepsis, and it is therefore notable that the peptides significantly dampen preferably the pro-inflammatory cytokine response. With respect to the anti-inflammatory cytokine IL-10, it is notable that a transient increase was observed in the early stages of LPS-shock. This induction was neither observed *in vivo* at later stages, nor in the *in vitro* models. Considering that IL-10 is pleiotropic and suppresses macrophage and dendritic cell functions, thereby limiting Th1 and Th2 effector responses [48], further studies are warranted in order to explore the exact mechanisms by which GKY25 modulate this particular cytokine. In addition to these immunomodulating effects, particularly GKY25 exert anticoagulative effects by blocking the contact system and directly modulating TF-mediated coagulation, representing a previously unknown capacity of this peptide. From a biological point of view this may reflect the fact that the initial procoagulative effects of thrombin are followed by the subsequent generation of anticoagulative fragments produced in response to proteases such as neutrophil elastase. Since similar C-terminal thrombin derived fragments are present in fibrin and wound fluid [26], these peptides anticoagulative effects may thus constitute a novel and logical feedback loop balancing coagulation and fibrin formation *in vivo* during e.g. wounding. In this context, it is interesting to note that the LPS-binding peptide WFF25, of similar charge and amino acid composition, albeit prolonging aPTT at higher doses, showed significantly less inhibition of TF expression on LPS-stimulated monocytes. This observation is compatible with the crucial role of TF for activation of coagulation *in vivo*, and therefore, the inhibitory effects by GKY25 on TF are of importance due to this factor's pivotal role as mediator between coagulation and inflammation. For example, mice with low levels of TF have been found to display decreased mortality in animal endotoxemia models [13], [49], an observation which is compatible with the observed therapeutic effects of GKY25 in this work. Disseminated intravascular coagulation (DIC) is a frequent complication of sepsis [30]. An indicator for DIC is thrombocytopenia [34] which was dampened in the peptide treated groups in both *in vivo* models. Activation of coagulation, inhibition of fibrinolysis, and consumption of coagulation inhibitors lead to a procoagulant state resulting in fibrin deposition in the microvasculature as observed in the lungs from mice subjected to LPS-mediated shock and *P. aeruginosa* infection. As a consequence, microvascular thrombosis contributes to promotion of organ dysfunction. Furthermore, excessive contact activation leads to the release of the pro-inflammatory peptide bradykinin and a subsequent induction of inflammatory reactions, which contribute to serious complications such as hypotension and vascular leakage [50], [51]. Therefore peptides, which in a biologically relevant context modulate several pathways, including inflammation and coagulation as demonstrated for these thrombin-derived peptides, are of interest in developing future peptide-based treatments for patients presenting with an excessive activation of these pathways, such as seen in sepsis.

## Supporting Information

Table S1 **Cytokine data of mice treated with buffer or peptides alone.** Mice were injected (ip) with buffer or 0.5 mg of peptide, and cytokines were analyzed after 20 h. Data are presented as mean ± SEM (buffer n = 6, GKY25 n = 6; HVF18 n = 2).
(PDF)

Figure S1 **Effects of GKY25 on macrophage activation by pro-inflammatory stimuli.** RAW-Blue macrophages were stimulated with 10 ng/ml TNF-α, 25 µg/ml zymosan, or 100 ng/ml ODN1826 in the absence or presence of GKY25. Cell activation was determined after 20 h using the QUANTI-Blue assay. Data are presented relative to controls without peptide and the mean ± SEM is shown (n = 5).
(TIF)

Figure S2 **Peptide effects on clotting parameters.** The activated partial thrombin time (aPTT) and prothrombin time (PT), respectively, were measured after addition of GKY25, HVF18, and WFF25 at various concentrations to human citrate plasma (n = 3, mean ± SEM is presented).
(TIF)

Figure S3 **Analysis of TF-expression of human monocytes.** Human monocytes were stimulated with 100 ng/ml *E. coli* LPS, in presence or absence of peptides (10 µM GKY25/WFF25 or 40 µM HVF18). After 18–20 h, CD14 and TF expression were determined by FACS. Representative FACS plots are shown (n = 3).
(TIF)

Figure S4 **Effects of WFF25 on cytokines during LPS-induced shock.** C57BL/6 mice were injected intraperitoneally (i.p.) with *E. coli* LPS (18 mg/kg), followed by i.p. administration of WFF25 (0.5 mg). The indicated cytokines were analyzed in plasma (n = 6–8/group).
(TIF)

Figure S5 **Histology score of lung tissues.** C57BL/6 mice were injected intraperitoneally (i.p) with *E. coli* LPS (18 mg/kg), followed by i.p. administration of GKY25 or HVF18 (0.5 mg). Scoring of hematoxylin-eosin stained lung sections (20 h efter LPS-challenge), according to the indicated criteria, was thereafter performed. Values are presented as mean ± SEM.
(TIF)

Figure S6 **Analyses of effects of GKY25 given alone.** (A) Analyses of animal lungs 20 h after i.p. injection of buffer or GKY25 (0.5 mg). Scanning electron micrographs show representative mouse lung sections (scale bar: 50 µm). (B–C) Subcutaneous administration of 0.5 mg GKY25 or buffer at 0 h and 6 h. (B) Determination of platelets after 12 h. (C) Measurement of activated partial thromboplastin time (aPTT) and prothrombin time (PT) in mouse plasma after 12 h (n = 6/group).
(TIF)

Figure S7 **Kinetics of** ***P. aeruginosa*** **infection in C57BL/6 and effects on cytokines.** Mice were infected i.p. with *P. aeruginosa* (5×10^8^ cfu/ml) and GKY25 (0.5 mg) was administrated s.c. 1 h after infection. (A) Bacterial counts in the indicated organs were analyzed after a time period of 4 h, 8 h and 12 h. (B) In parallel; the indicated cytokines were analyzed in plasma. (C) Effects on platelets counts are shown. (n = 5–7/group).
(TIF)

Figure S8 **Kinetics of cytokines during** ***P. aeruginosa*** **infection and effects of GKY25.** Mice were infected i.p. with a high dose of *P. aeruginosa* (5×10^9^ cfu/ml) and GKY25 (0.5 mg) was administrated s.c. One h after infection, the indicated cytokines were analyzed in plasma after the indicated time periods (n = 4/group).
(TIF)

Materials and Methods S1 **Quanti Blue Assay.**
(DOCX)

Materials and Methods S2 **Histology scores.**
(DOCX)
