# Supplementary material for: Host Defense Peptides of Thrombin Modulate Inflammation and Coagulation in Endotoxin-Mediated Shock and Pseudomonas aeruginosa Sepsis
Source: PLoS One. 2012 Dec 13;7(12):e51313. doi: 10.1371/journal.pone.0051313 (PMC3521733; doi:10.1371/journal.pone.0051313)
Supplement: Table S1 — Cytokine data of mice treated with buffer or peptides alone. Mice were injected (ip) with buffer or 0.5 mg of peptide, and cytokines were analyzed after 20 h. Data are presented as mean ± SEM (buffer n = 6, GKY25 n = 6; HVF18 n = 2). (PDF) [file pone.0051313.s001.pdf]

	**IL-6 (ng/ml)**	**TNF-α (ng/ml)**	**MCP-1 (ng/ml)**	**IFN-γ (ng/ml)**	**IL-10 (ng/ml)**
**Buffer**	0.0067 ± 0.0002	0.0355 ± 0.0018	0.0081 ± 0.003	0.0018 ± 0.001	0.00062 ± 0.0006
**GKY25**	0.0079 ± 0.0053	0.0029 ± 0.0010	0.1512 ± 0.095	0.0002 ± 0.0001	0.0005 ± 0.0006
**HVF18**	0.0015 ± 0.0003	0.0041 ± 0.0003	0.0136 ± 0.003	0.0002 ± 0.0001	0.0042 ± 0.0006

.
