# Supplementary figures and images for: Host Defense Peptides of Thrombin Modulate Inflammation and Coagulation in Endotoxin-Mediated Shock and Pseudomonas aeruginosa Sepsis
Source: PLoS One. 2012 Dec 13;7(12):e51313. doi: 10.1371/journal.pone.0051313 (PMC3521733; doi:10.1371/journal.pone.0051313)

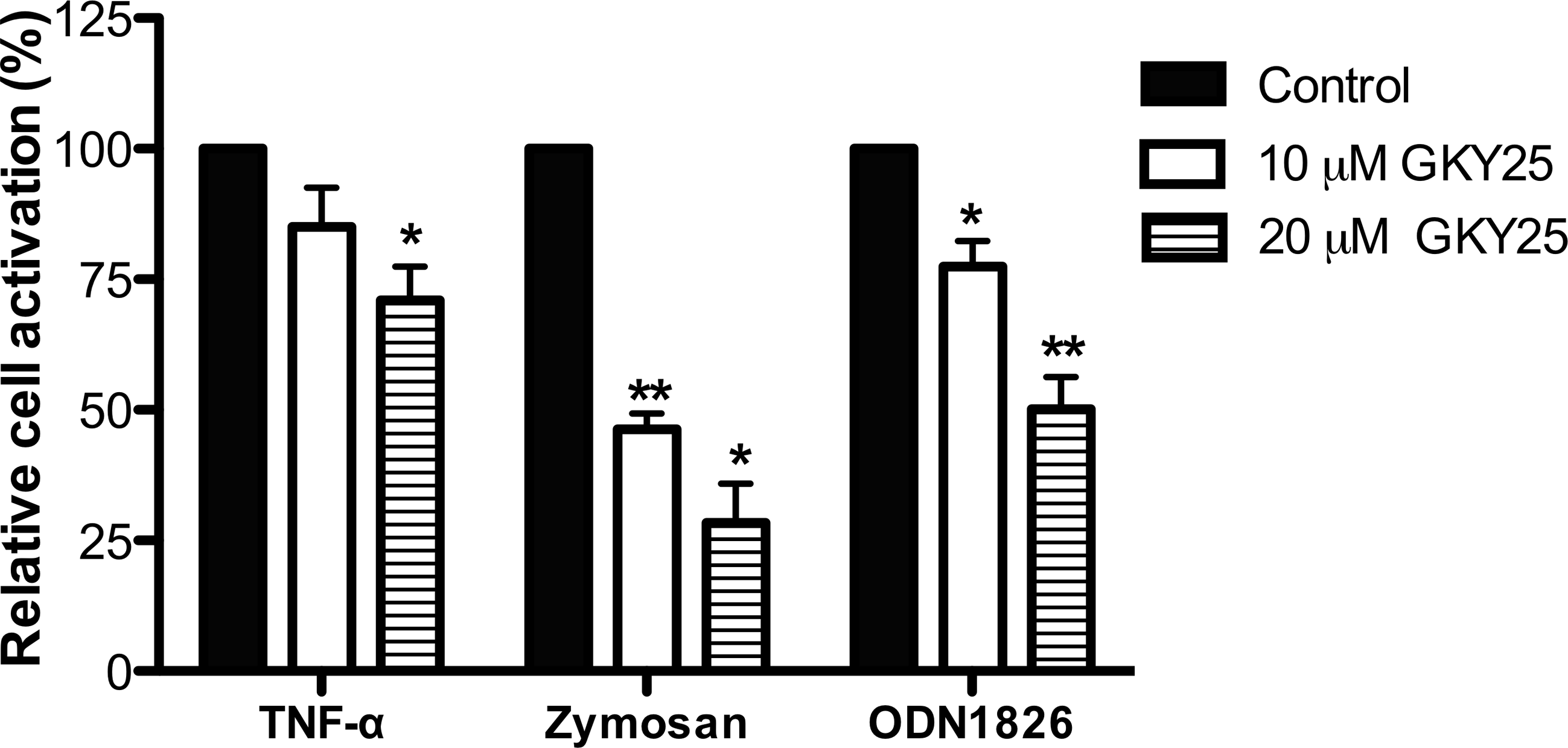

Supplement: Figure S1 — Effects of GKY25 on macrophage activation by pro-inflammatory stimuli. RAW-Blue macrophages were stimulated with 10 ng/ml TNF-α, 25 µg/ml zymosan, or 100 ng/ml ODN1826 in the absence or presence of GKY25. Cell activation was determined after 20 h using the QUANTI-Blue assay. Data are presented relative to controls without peptide and the mean ± SEM is shown (n = 5). (TIF) [file pone.0051313.s002.tif]

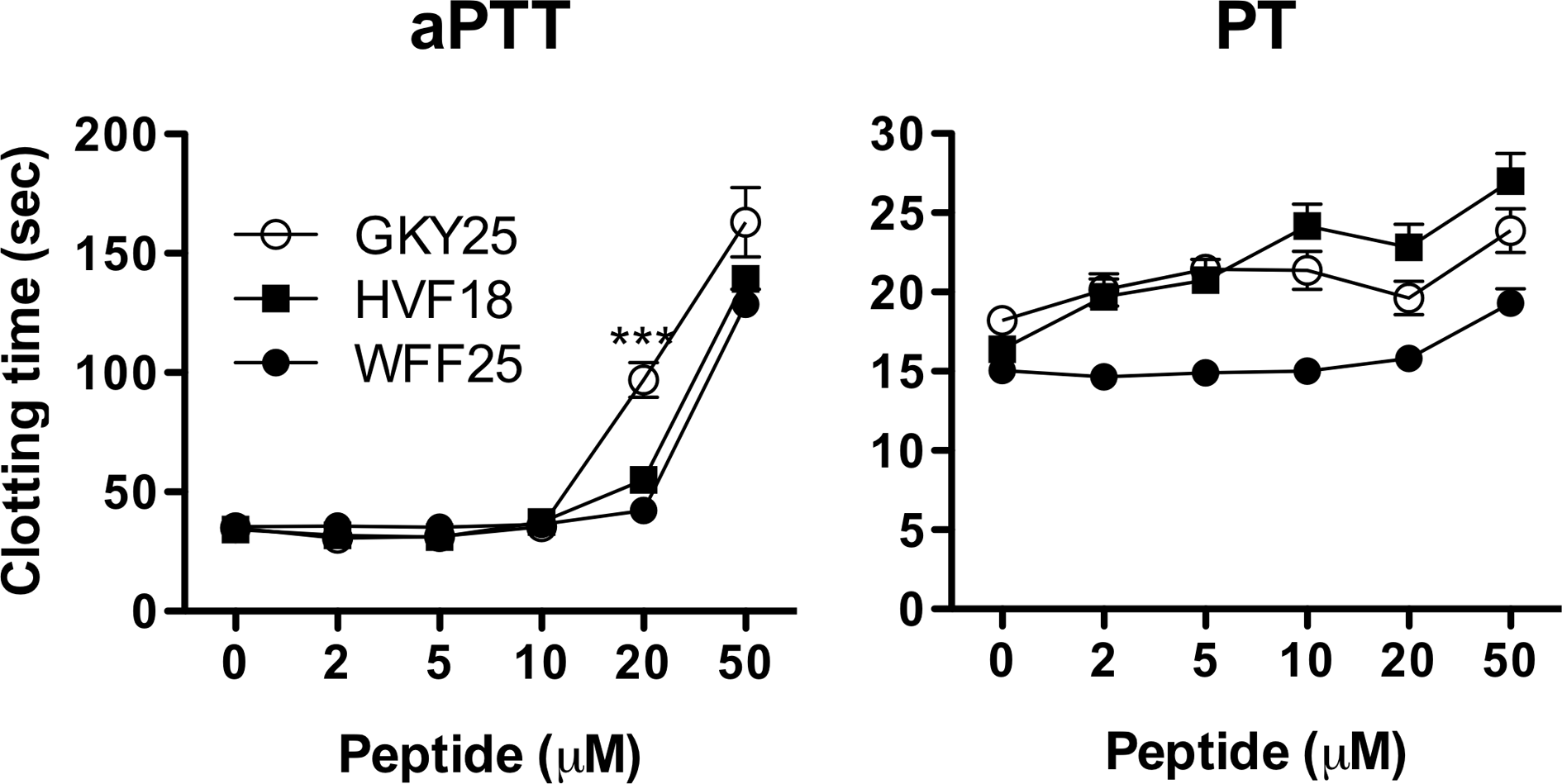

Supplement: Figure S2 — Peptide effects on clotting parameters. The activated partial thrombin time (aPTT) and prothrombin time (PT), respectively, were measured after addition of GKY25, HVF18, and WFF25 at various concentrations to human citrate plasma (n = 3, mean ± SEM is presented). (TIF) [file pone.0051313.s003.tif]

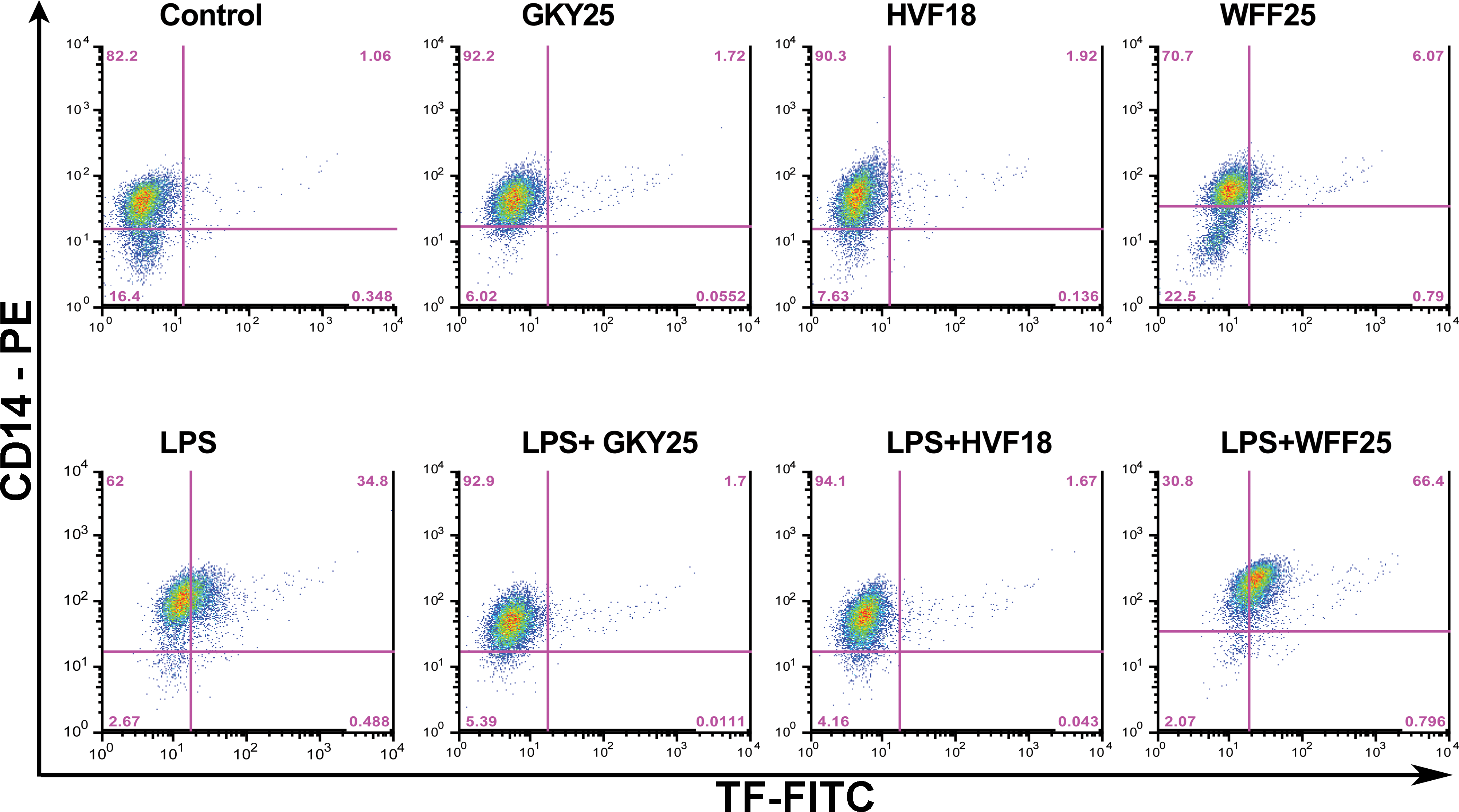

Supplement: Figure S3 — Analysis of TF-expression of human monocytes. Human monocytes were stimulated with 100 ng/ml E. coli LPS, in presence or absence of peptides (10 µM GKY25/WFF25 or 40 µM HVF18). After 18–20 h, CD14 and TF expression were determined by FACS. Representative FACS plots are shown (n = 3). (TIF) [file pone.0051313.s004.tif]

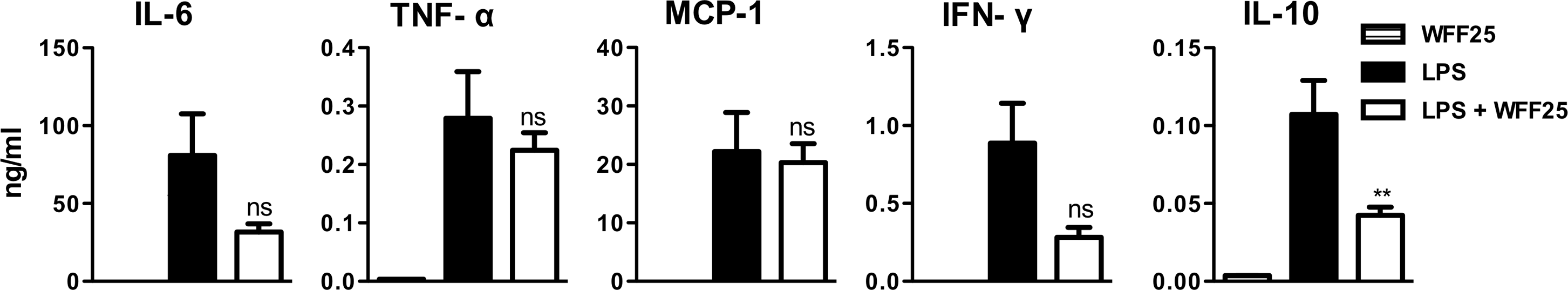

Supplement: Figure S4 — Effects of WFF25 on cytokines during LPS-induced shock. C57BL/6 mice were injected intraperitoneally (i.p.) with E. coli LPS (18 mg/kg), followed by i.p. administration of WFF25 (0.5 mg). The indicated cytokines were analyzed in plasma (n = 6–8/group). (TIF) [file pone.0051313.s005.tif]

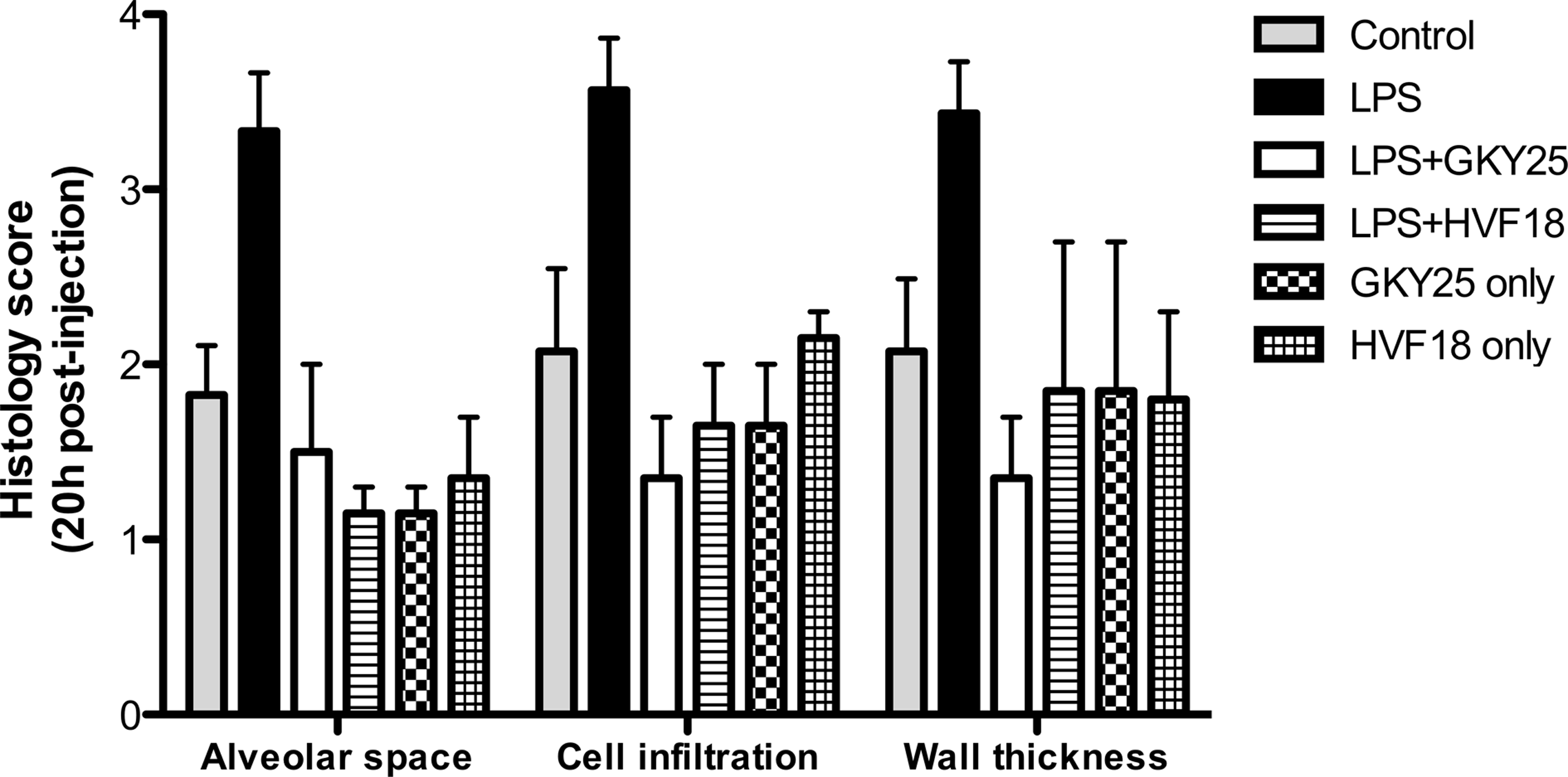

Supplement: Figure S5 — Histology score of lung tissues. C57BL/6 mice were injected intraperitoneally (i.p) with E. coli LPS (18 mg/kg), followed by i.p. administration of GKY25 or HVF18 (0.5 mg). Scoring of hematoxylin-eosin stained lung sections (20 h efter LPS-challenge), according to the indicated criteria, was thereafter performed. Values are presented as mean ± SEM. (TIF) [file pone.0051313.s006.tif]

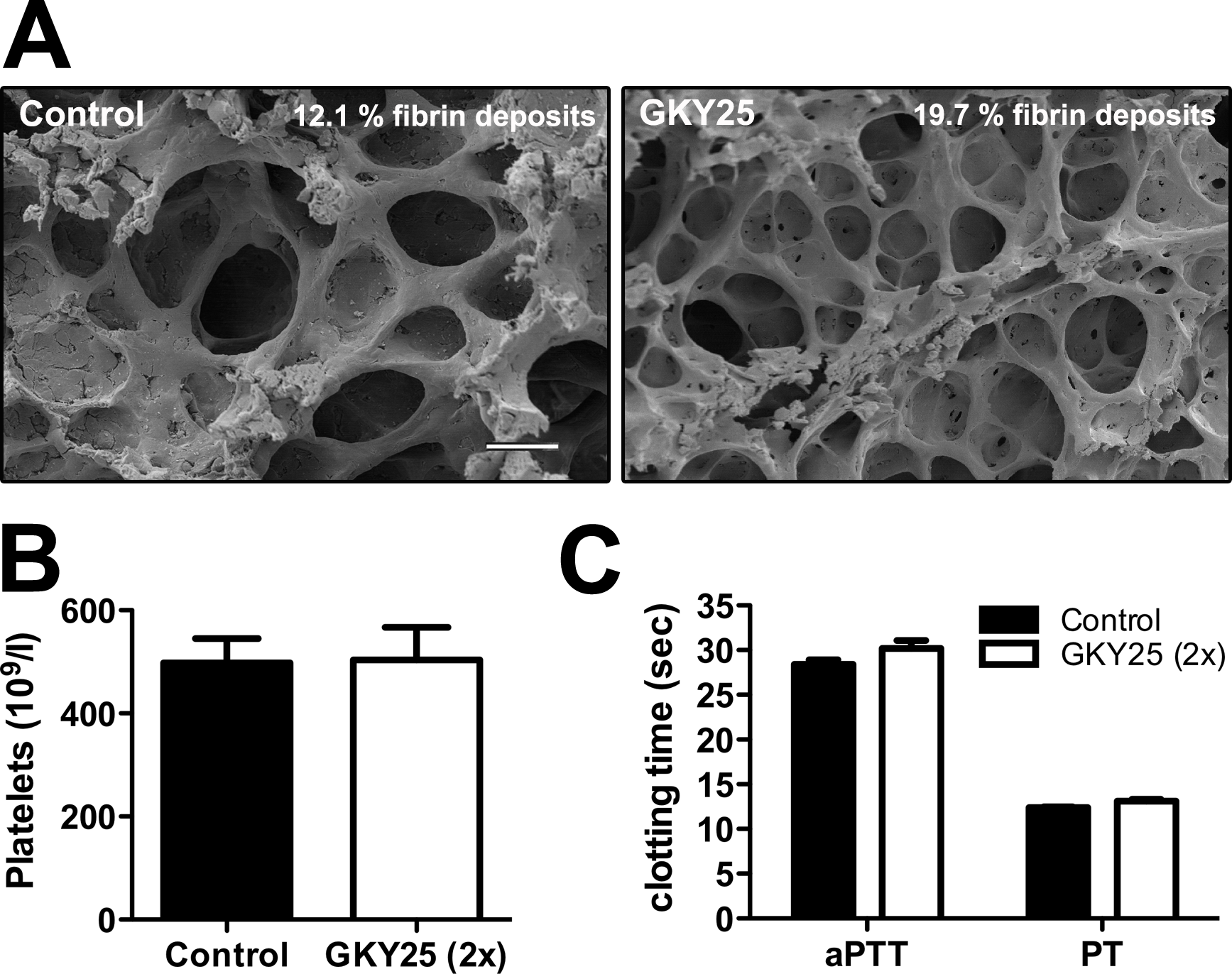

Supplement: Figure S6 — Analyses of effects of GKY25 given alone. (A) Analyses of animal lungs 20 h after i.p. injection of buffer or GKY25 (0.5 mg). Scanning electron micrographs show representative mouse lung sections (scale bar: 50 µm). (B–C) Subcutaneous administration of 0.5 mg GKY25 or buffer at 0 h and 6 h. (B) Determination of platelets after 12 h. (C) Measurement of activated partial thromboplastin time (aPTT) and prothrombin time (PT) in mouse plasma after 12 h (n = 6/group). (TIF) [file pone.0051313.s007.tif]

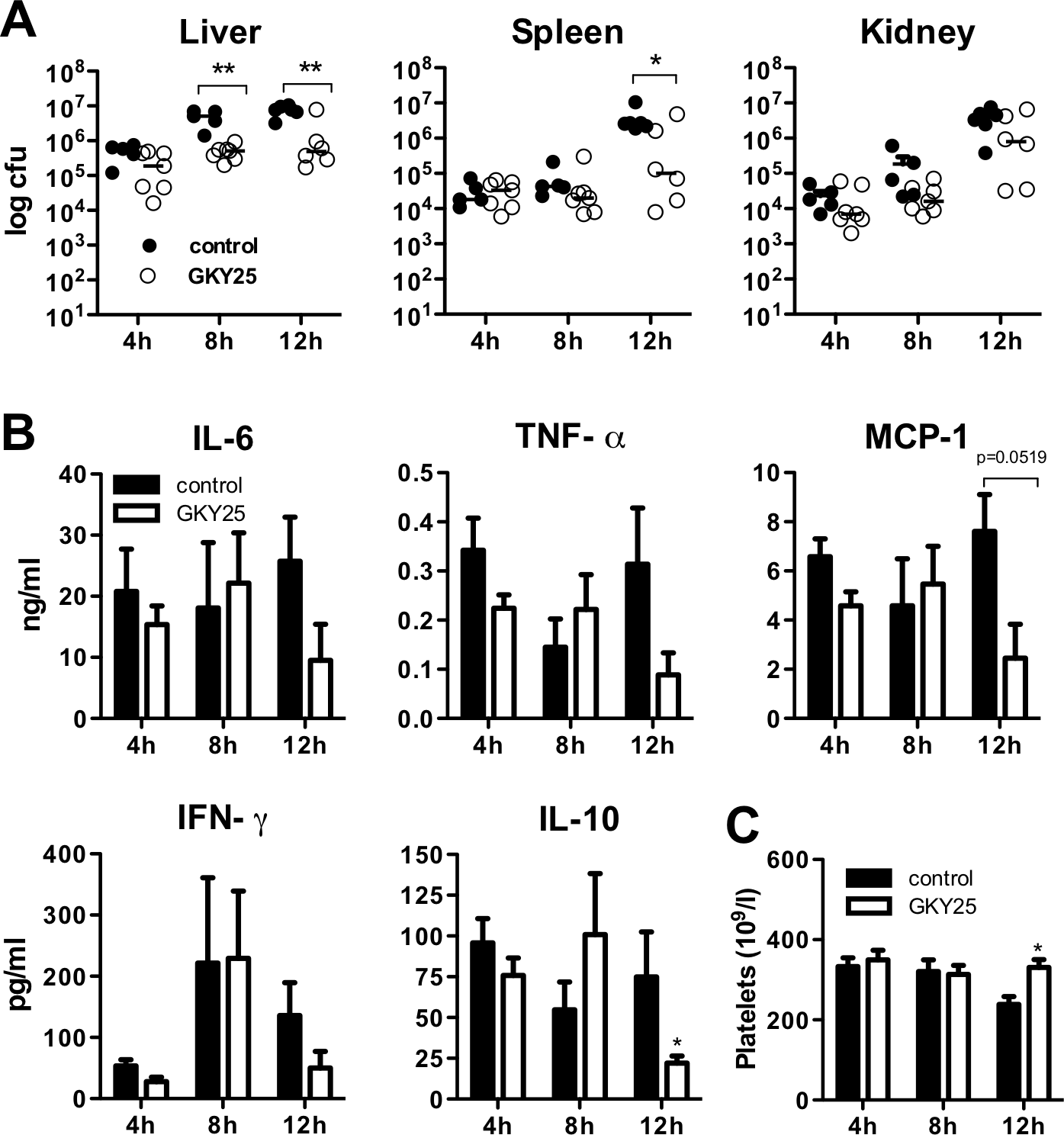

Supplement: Figure S7 — Kinetics of P. aeruginosa infection in C57BL/6 and effects on cytokines. Mice were infected i.p. with P. aeruginosa (5×10^8^ cfu/ml) and GKY25 (0.5 mg) was administrated s.c. 1 h after infection. (A) Bacterial counts in the indicated organs were analyzed after a time period of 4 h, 8 h and 12 h. (B) In parallel; the indicated cytokines were analyzed in plasma. (C) Effects on platelets counts are shown. (n = 5–7/group). (TIF) [file pone.0051313.s008.tif]

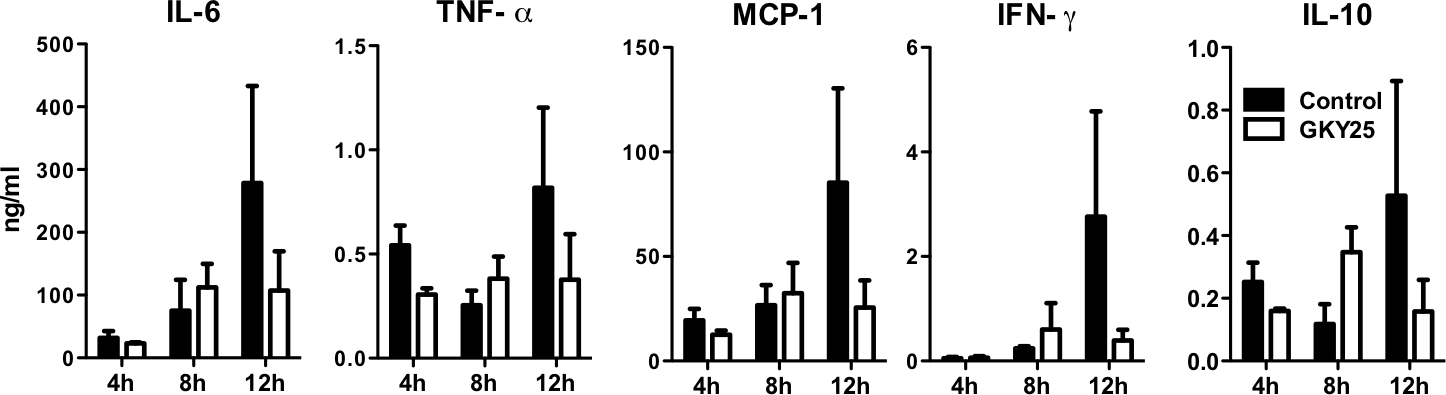

Supplement: Figure S8 — Kinetics of cytokines during P. aeruginosa infection and effects of GKY25. Mice were infected i.p. with a high dose of P. aeruginosa (5×10^9^ cfu/ml) and GKY25 (0.5 mg) was administrated s.c. One h after infection, the indicated cytokines were analyzed in plasma after the indicated time periods (n = 4/group). (TIF) [file pone.0051313.s009.tif]
