# Supplementary material for: Host Defense Peptides of Thrombin Modulate Inflammation and Coagulation in Endotoxin-Mediated Shock and Pseudomonas aeruginosa Sepsis
Source: PLoS One. 2012 Dec 13;7(12):e51313. doi: 10.1371/journal.pone.0051313 (PMC3521733; doi:10.1371/journal.pone.0051313)
Supplement: Materials and Methods S1 — Quanti Blue Assay. (DOCX) [file pone.0051313.s010.docx]

**Materials and Methods S1**

**Quanti Blue Assay -** The mouse macrophage cell line RAW-Blue (InvivoGen) derived from RAW 264.7 cells was cultured in Dulbecco´s modified Eagle medium (DMEM; PAA-Laboratories) supplemented with 10% (v/v) heat-inactivated fetal bovine serum (FBS) (Invitrogen) and 1% (v/v) Antibiotic-Antimycotic solution (AAS) (Invitrogen) and 200 μg/ml Zeocin (InvivoGen). RAW-Blue (1×10^6^ /ml) in phenol red-free DMEM supplemented with 10% (v/v) FBS and 1% (v/v) AAS were seeded in 96-wells tissue culture plates (Nunc). Following 20 h of incubation to allow adherence, cells were stimulated with recombinant mouse TNF-α (10 ng/ml) (eBioscience), zymosan (25 μg/ml) (Sigma-Aldrich) or ODN1826 (100 ng/ml) (Invivogen), with or without the addition of GKY25. Cell activation was determined after 20 h of incubation by using the Quanti-Blue^TM^ assay according to the manufacturers instructions (Invivogen).
