# Supplementary material for: Host Defense Peptides of Thrombin Modulate Inflammation and Coagulation in Endotoxin-Mediated Shock and Pseudomonas aeruginosa Sepsis
Source: PLoS One. 2012 Dec 13;7(12):e51313. doi: 10.1371/journal.pone.0051313 (PMC3521733; doi:10.1371/journal.pone.0051313)
Supplement: Materials and Methods S2 — Histology scores. (DOCX) [file pone.0051313.s011.docx]

**Materials and Methods S2**

**Histology scores -** C57BL/6 mice were injected i.p. with buffer or *E. coli* LPS (18 mg/kg, Sigma-Aldrich) followed by i.p. injection of GKY25 or HVF18 (0.5 mg/kg). Hematoxylin-eosin stained lung sections of the animals were blindly scored by three independent observers. Reduction in alveolar space, increase in cell infiltration, and thickness of alveolar septa (cell wall thickness) was determined (score 1- 4; 1=no change (normal), 2=minor, 3=medium, 4=significant change). At least 5 different view fields were observed from each section.
